# Suppression of Inflammatory and Fibrotic Signals by Cinnamon (*Cinnamomum cassia*) and Cinnamaldehyde in Cyclophosphamide-Induced Overactive Bladder in Mice

**DOI:** 10.1155/2021/5205759

**Published:** 2021-12-22

**Authors:** Lih-Lian Chen, Mei-Hsien Lee, Chia-Lin Chang, Kuo-Tong Liou, Shu-Hsiang Liu, Chang-Ming Chern, Hui-I. Chen, Yuh-Chiang Shen, Yea-Hwey Wang

**Affiliations:** ^1^Ph.D. Program in Clinical Drug Development of Herbal Medicine, College of Pharmacy, Taipei Medical University, Taipei City, Taiwan; ^2^Department of Traditional Chinese Medicine, En Chu Kong Hospital, New Taipei City, Taiwan; ^3^Graduate Institute of Pharmacognosy, College of Pharmacy, Taipei Medical University, Taipei City, Taiwan; ^4^Center for Reproductive Medicine & Sciences, Taipei Medical University Hospital, Taipei City, Taiwan; ^5^Research Institute of Biotechnology, Hungkuang University, Taichung City, Taiwan; ^6^Department of Medicine, Mackay Medical College, New Taipei City, Taiwan; ^7^Department of Chinese Medicine, Tri-Service General Hospital, National Defense Medical Center, Taipei City, Taiwan; ^8^School of Nursing, College of Nursing, National Taipei University of Nursing and Health Sciences, Taipei City, Taiwan; ^9^Taipei Municipal Gan-Dau Hospital, Taipei City, Taiwan; ^10^Division of Neurovascular Disease, Neurological Institute, Taipei Veterans General Hospital, Taipei City, Taiwan; ^11^Institute of Brain Science, School of Medicine, National Yang-Ming University, Taipei City, Taiwan; ^12^National Research Institute of Chinese Medicine, Ministry of Health and Welfare, Taipei City, Taiwan

## Abstract

Cinnamon (*Cinnamomum cassia*) is a well-known traditional Chinese medicine used to treat nocturia by tonifying and warming the kidney. Our recent clinical study found that overactive bladder (OAB) patients treated with cinnamon powder (CNP) patches exhibited significantly ameliorated OAB symptoms without significant side effects, but the mechanism of action is unclear. To explore the beneficial effects and action mechanisms of CNP and its major active component cinnamaldehyde (CNA) in an OAB-related murine model, cyclophosphamide- (CYP-) induced OAB injury was performed on male *ICR mice* in the presence or absence of CNP and CNA, as well as solifenacin, a clinical drug for OAB as a reference. Twenty-four-hour micturition patterns (frequency of urination and volume of urine per time), as well as histopathological examination, immunohistochemistry (IHC), and Western blotting of the bladder, were analyzed for mechanism elucidation. Administration of CYP (300 mg/kg, i.p.) induced typical OAB pathophysiological changes, including increased frequency of urination and reduced volume of urine. CYP-induced mice displayed strong edema of the bladder and hemorrhagic cystitis, accompanied by loss of normal corrugated folds and decreased muscarinic receptors (M2/M3) in the urothelium, and disordered/broken structures of the lamina propria and detrusor. These changes were correlated with increased leukocyte (CD11b) infiltration colocalized with inflammatory (pp65 NF*κ*B, macrophage migration inhibitory factor (MIF)*/Toll-like receptor 4 (*TLR4)) and fibrotic (stem cell factor (SCF)/c-Kit, *α*-*smooth muscle actin* (*α*-SMA)/*β*-catenin) signals. Treatment with CNP (600 mg/kg, p.o.) and CNA (10–50 mg/kg, p.o.), but not solifenacin (50 mg/kg), 30 min after CYP induction significantly ameliorated CYP-induced dysfunction in micturition patterns and pathophysiological changes. CNP and CNA further suppressed MIF/TLR4-associated inflammatory and SCF/c-Kit-related fibrotic signaling pathways. Our findings indicate that suppression of inflammatory and fibrotic signals contributes to the crucial mechanism in the improvement of CYP-induced OAB by CNP and CNA.

## 1. Introduction

Overactive bladder (OAB) is a disorder in which the bladder spasms and causes a sudden and strong urge to urinate accompanied by or without urge incontinence; sometimes, these urges can lead to events of uncontrolled urine leakage. The International Continence Society has defined OAB as a “symptom syndrome suggestive of lower urinary tract dysfunction” [1]. The incidence of OAB is 16.9% in women and 16% in men aged >18 years [2] and increases to 43.1% in women and 27.2% in men aged >40 years [3]. Besides age, risk factors for OAB include race/ethnicity, central or peripheral neuronal diseases (such as multiple sclerosis, stroke, dementia, and spinal cord injury), medications (such as anticholinergics, diuretics, alpha-adrenergic agonists, beta-adrenergic antagonists, and sedatives), pregnancy, and vaginal childbirth (increased risk of incontinence) [4].

Previous studies revealed that more inflammation-related substances are found in the bladders of OAB patients than in others. Afferent nerve terminals are sensitized directly by mediators including histamine, nerve growth factor (NGF) [5], prostaglandin E_2_ (PGE_2_) [6], cyclophosphamide [7], and those released from mast cells [8]. Prolonged hypersensitivity to inflammatory mediators of afferent neurons will generate a frequent desire to urinate and lead to bladder overactivity [9, 10]. First-line treatment by lifestyle modification (e.g., changing the drinking strategy) and pelvic floor muscle rehabilitation are recommended for mild OAB patients. Competitive cholinergic M3 receptor antagonists, such as solifenacin, are suggested as second-line treatment [11]. However, solifenacin causes many side effects, including dry mouth in 10.9–27.6% of patients and constipation in 5.4–13.4% of patients [12]. These side effects lead to low compliance and discontinued OAB medication in 21.1% of patients and thus unmet treatment expectations [13]. Therefore, it is critical to develop a new medication for relieving the symptoms of OAB effectively with fewer side effects.

Cinnamon (*Cinnamomum cassia* (L.) J. Presl) belongs to the Lauraceae family. The powdered bark of cinnamon (CNP) is a popular traditional herbal drug. It also has been applied to nocturia by tonifying and warming the kidney in Traditional Chinese Medicine (TCM) [14]. Current research has revealed that cinnamon exhibits anti-inflammatory activity and acts as an antioxidant, ameliorates neurological disorders and cardiovascular disease, and has antidiabetic activity, antimicrobial activity, anticancer activity, and lipid-lowering effects [15]. The bark of cinnamon contains around 60–85% of cinnamaldehyde [16]. Cinnamaldehyde (CNA), also (2E)-3-phenylprop-2-enal, the major natural oil isolated from cinnamon, is made from extracting the inner bark of the trunks of cinnamon trees [17]. CNA has been reported to improve stress urinary incontinence with anti-inflammatory potential [18]. Our recent clinical study found that treatment of OAB patients with CNP patches could significantly ameliorate the OAB symptoms without significant side effects [19]. However, no studies have evaluated the advantageous effects and molecular mechanisms of CNP and its major active component CNA in an OAB-related model. In this study, we used a mouse model of cyclophosphamide-induced OAB to explore the protective effects of CNP and CNA as well as their underlying mechanisms of action. We found that CNP and CNA have an OAB-ameliorative effect. These advantageous effects of CNP and CNA could be explained by their anti-inflammatory and antifibrotic effects, leading to amelioration of micturition dysfunction.

## 2. Materials and Methods

### 2.1. Reagent Preparation and Administration

**Figure 1:**
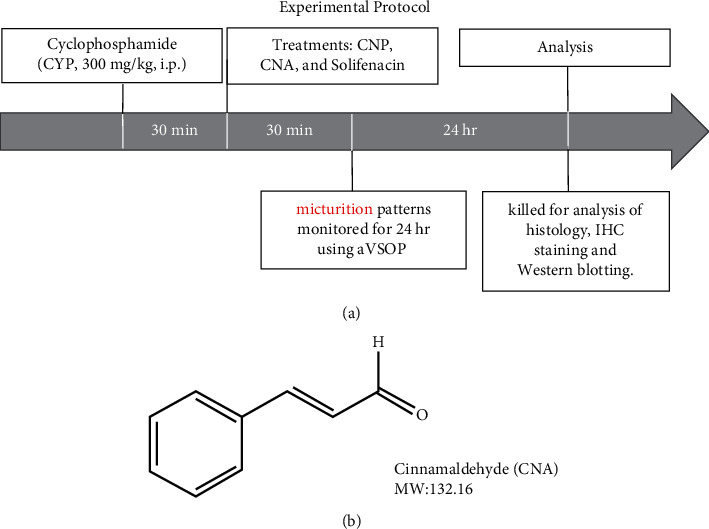
(a) General experimental protocol. (b) Chemical structure of cinnamaldehyde (CNA).

The crude bark powder of cinnamon (CNP) was purchased from an international herbal drug company (Shen Chang GMP-Certified Pharmaceutical Co., Taoyuan, Taiwan; batch number: BA191012A; Ministry of Health and Welfare (Taiwan), Manufacturing no. 053990). It was dissolved in dimethyl sulfoxide (DMSO) to 60 mg/ml stock solution and stored at −20°C. The chemical constituents of CNP were examined using ultraperformance liquid chromatography diode array detector tandem mass spectrometry (UPLC-DAD-MS/MS). Relative contents (mg/g dry weight) of the chemical constituents of CNP including cinnamaldehyde (CNA), cinnamic acid, 2-methoxyamaldehyde, coumarin, and cinnamyl alcohol were determined by UPLC-DAD-MS/MS to be 5.21 ± 0.07, 1.80 ± 0.02, 0.99 ± 0.01, 0.69 ± 0.01, and 0.16 ± 0.00. Cinnamaldehyde (CNA, natural, >95% (HPLC), Figure 1(b)) and a reference clinical compound for OAB, solifenacin (≥98% (HPLC); a M3 muscarinic antagonist) and HC-030031 (≥98% (HPLC); a TRPA1 antagonist) were purchased from a chemical and drug company (Sigma-Aldrich) and dissolved in distilled water to produce 10−100 mg/ml stock solution, which was stored at −20°C. All treatments were administrated 30 min after cyclophosphamide (CYP, ≥90% purity, from Sigma-Aldrich) treatment protocol (Figure 1(a)). Different animal treatment groups were arranged by randomized selection of a vehicle (saline), control or test drug (CNP and CNA) (Figure 1(b)), or reference drug (solifenacin) by an investigator unconnected to the study to reduce treatment bias. The results were decoded only at the study end.

### 2.2. Animal Preparation and CYP-Induced Cystitis-Related Overactive Bladder (OAB) Murine Model

The Guide for the Care and Use of Laboratory Animals [20] was followed for all animal procedures and protocols conducted in this study. The Animal Research Committee of the National Research Institute of Chinese Medicine (NRICM) also reviewed and approved these protocols (approval number: NRICM-IACUC-108-912-1). A mixture of zolazepam (40 mg/kg, i.p.) and xylazine (10 mg/kg, i.p.) was used to anesthetize male ICR mice (28–30 g) (National Laboratory Animal Breeding and Research Center, Taipei, Taiwan). Acute urinary toxicity, referred to as a cystitis-related overactive bladder (OAB), was induced in mice by a single injection of cyclophosphamide (CYP, 300 mg/kg, i.p.) as previously described [21].

### 2.3. Experimental Design for the Intervention of Crude Bark Powder of Cinnamon (CNP), Cinnamaldehyde (CNA), and Solifenacin

Normally, mice were randomly allocated to seven groups: (1) a sham control group (control, *n* = 30) without cyclophosphamide (CYP) induction; (2) one cinnamon powder (CNP) (at 600 mg/kg, 30 min after CYP induction, p.o.) treatment group with CYP (CYP + CNP, *n* = 30); (3, 4, and 5) three cinnamaldehyde (CNA) (at 10 mg/kg, 50 mg/kg or 100 mg/kg, 30 min after CYP induction, p.o.) treatment groups with CYP (CYP + CNA (10), CYP + CNA (50), or CYP + CNA (100), *n* = 30 for each dose); (6) one solifenacin (at 50 mg/kg [22], 30 min after CYP, p.o.) treatment group with CYP (CYP + solifenacin, *n* = 20); and (7) a vehicle (normal saline with 0.1% of DMSO) group with CYP (CYP + Veh, *n* = 30). The experimental protocol is revealed in Figure 1(a).

### 2.4. Evaluation of 24-Hour Micturition Pattern, Hemorrhagic Cystitis, and Inflammation-Related Edema after Acute CYP-Induced OAB

Sixty minutes after CYP-induced OAB, micturition patterns of mice were analyzed by an automated Voided Stain on Paper (aVSOP) method [21, 23]. Briefly, urine stains (i.e., micturition time (frequency) and volume) within 24 hours after acute CYP-induced OAB were counted and traced. A standard curve was created first using the correlation between normal saline volume (ranging from 5 to 640 *μ*l) and stained area (cm × cm) (*r*2 = 0.9934). Then, we used the standard curve formula to calculate the micturition volume (*μ*l) of CYP-induced OAB mice from different groups. Mice were kept in a 75 × 160 × 75 mm (height × depth × width) cage with a 12 hr light and 12 hr dark cycle and free access to water and food. The amounts of water and food taken were not counted. After analysis of the micturition patterns, mice were quickly decapitated and killed under deep anesthesia. The whole bladder was rapidly removed, emptied of remaining urine, cleaned of connective tissue, and weighed and photographed for determination of inflammation-related edema and hemorrhagic cystitis, immunohistochemical (IHC) staining, and Western blotting.

### 2.5. Histopathological Analysis

For histopathological analysis, 6–10 consecutive bladder sections (thickness, 20 *μ*m) from the frozen embedded entire bladders of different treatment groups were collected and cut at the same level near the middle of the organ. Following fixation with 4% (v/v) formalin and staining with hematoxylin-eosin (H&E), the extent of changes in the morphological structure of the urothelium (U), lamina propria (L), and bladder detrusor (D), damaged areas with significant loss of the normal corrugated folds of the urothelium, and tissue arranged in disorder and fracture (fragmented) with swollen/empty spaces were photographed under a microscope (10× or 20×). These slides were examined later by three investigators who were blind to animal groups and who scored them as negative (0), mild (1), moderate (2), and severe (3) for hemorrhagic cystitis and damaged tissue arranged in disorder/fragmented and swollen with empty spaces.

### 2.6. Immunohistochemical (IHC) Staining

Twenty-four hours after acute CYP-induced OAB, the bladders were prepared for confocal analysis according to our previous report [24]. Briefly, 6–10 consecutive bladder sections (thickness, 20 *μ*m) from the frozen embedded entire bladders of each group were collected and cut at the same levels near the middle of the organ. After fixation, permeabilization, and blocking, the bladder slices were randomly selected for incubation with proper primary antibodies against muscarinic M2 and M3 (1 : 200) (US Biological, CO, USA), CD11b and *β*-catenin (1 : 50, Abcam, Cambridge, UK), pp65 NF*κ*B (1 : 50, BD PharMingen, San Diego, CA, USA), MIF and TLR4 (1 : 100, GeneTex, Irvine, CA, USA), SCF (1 : 50, Santa Cruz Biotechnology, Inc., Santa. Cruz, CA, USA), c-Kit (1 : 200, Invitrogen, Frederick, MD, USA), TRPA1 (1 : 200, Novus Biologicals, USA), and *α*-SMA (1 : 500, GeneTex, Irvine, CA, USA) diluted in PBS with 3% albumin at 4°C overnight. After washing, all sections were incubated with Alexa Fluor® 488-, Alexa Fluor® 555-, or Alexa Fluor® 647-conjugated secondary antibodies (Cell Signaling Technology Inc., MA, USA). A mounting medium containing 4′,6-diamidino-2-phenylindole (DAPI) was used to mount all coverslips to counterstain the DNA in the nuclei. Then, all sections were examined using a laser-scanning confocal microscope (Zeiss LSM780, Carl Zeiss, Jena, Germany). The distribution areas of immunopositively stained cells were identified and quantified as the positive stained area percentages (%) of the whole images using imaging software (Zen 2011, black edition, Carl Zeiss MicroImaging GmbH, 1997–2011; AlphaEaseFC software, version 4.0, Alpha Innotech Corporation, CA, USA) across the entire field (10×) of selected images or after sampling in specific regions (such as urothelium) under high magnification (10×–20×) over 3–5 independent experiments.

### 2.7. Western Immunoblotting Analysis

We collected equal amounts of protein (20–40 *μ*g) from the whole bladders of treatment groups described above and subjected them to sodium dodecyl sulfate-polyacrylamide gel electrophoresis (8–12%) and electrotransfer to a hydrophobic polyvinylidene difluoride (PVDF) membrane, as described previously [24]. The PVDF membranes were blocked and incubated overnight at 4°C with a primary antibody against pp65 NF*κ*B (1 : 1000, BD PharMingen, San Diego, CA, USA), CD11b (1 : 500, Abcam, Cambridge, UK), SCF (1 : 1000, Santa Cruz Biotechnology, Inc., Santa. Cruz, CA, USA), muscarinic M3 (1 : 1000, US Biologicals, CO, USA) and TLR4 (1 : 1000, GeneTex, Irvine, CA, USA), TRPA1 (1 : 1000, Novus Biologicals, USA), or *β*-actin (1 : 1000, Millipore, CA, USA). After incubation of the samples with appropriately calculated secondary antibodies and developing with enhanced chemiluminescence (ECL) reagents, the Western blot on the membrane was visible on the X-ray films for quantification by imaging software (AlphaEaseFC, Alpha Innotech Corporation, USA).

### 2.8. Statistical Analysis

All data are presented as mean ± SEM (standard error of the mean). For multiple comparisons, one-way analysis of variance (ANOVA) followed by post hoc Student–Newman–Keuls (S-N-K) *t*-test was performed. Significance was set at *p* < 0.05.

## 3. Results

### 3.1. Ameliorative Effects of CNP and CNA on Micturition Dysfunction after Acute CYP-Induced OAB in Mice

**Figure 2:**
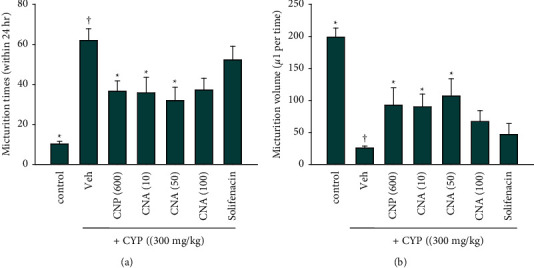
Micturition patterns of cyclophosphamide-induced OAB in mice. Male *ICR mice* were subjected to cyclophosphamide (CYP, 300 mg/kg)-induced OAB in the presence or absence of cinnamon powder (CNP) and cinnamaldehyde (CNA). Micturition patterns including (a) frequency (time within 24 hours) and (b) volume (*μ*l per time) within 24 hours were recorded using an automated Voided Stain on Paper (aVSOP) method. Animal groups: (1) sham control group (control, *n* = 10) without CYP induction; (2) one CNP (at 600 mg/kg, 30 min after CYP induction, p.o.) treatment group with CYP (CYP + CNP (600), *n* = 10); (3, 4, and 5) three CNA (at 10 mg/kg, 50 mg/kg, or 100 mg/kg, 30 min after CYP induction, p.o.) treatment groups with CYP (CYP + CNA (10) or CNA (50) or CNA (100), *n* = 10 for each dose); (6) one solifenacin (at 50 mg/kg, 30 min after CYP, p.o.) treatment group with CYP (CYP + solifenacin, *n* = 10); and (7) a vehicle (normal saline with 0.1% of dimethyl sulfoxide (DMSO)) group with CYP (CYP + Veh, *n* = 10). Data are presented as mean ± SEM. ^†^, ^*∗*^*p* < 0.05, compared with control or CYP + Veh group, respectively, by one-way ANOVA followed by S-N-K post hoc *t*-test for multiple comparisons.

To examine whether administration of CNP and CNA could ameliorate CYP-induced OAB, twenty-four-hour micturition patterns were evaluated using automated Voided Stain on Paper (aVSOP) after acute CYP-induced OAB. Intraperitoneal administration of CYP (300 mg/kg) in male ICR mice induced extensive urotoxicity that was associated with cystitis-related OAB. CYP dramatically increased the micturition frequency (times per 24 hours) (CYP 62 ± 6 vs. control 10 ± 1; Figure 2(a), one-way ANOVA, *p* < 0.05) and reduced the micturition volume (*μ*l per time) (CYP 25 ± 3 vs. control 199 ± 14, Figure 2(b), one-way ANOVA, *p* < 0.05). Oral treatment with CNP (600 mg/kg) and CNA (10–50 mg/kg) 30 min after CYP induction both significantly ameliorated the CYP-induced OAB related dysfunction in micturition frequency (times per 24 hours) (Figure 2(a), one-way ANOVA, *p* < 0.05) and micturition volume (*μ*l per time) (Figure 2(b), one-way ANOVA, *p* < 0.05). Particularly, CNA (at 50 mg/kg) showed the strongest protective effects in both the amelioration of micturition frequency and the micturition volume (Figure 2). A muscarinic M3 receptor antagonist (solifenacin) used in the clinic was included in this study as a reference drug. Our results showed that solifenacin did not produce significant improvement in abnormal micturition patterns (frequency and volume) in this CYP-induced OAB model (*p* > 0.05, Figure 2).

### 3.2. Effects of CNP and CNA on Histopathological Changes in Bladder Tissue after Acute CYP-Induced OAB in Mice

**Figure 3:**
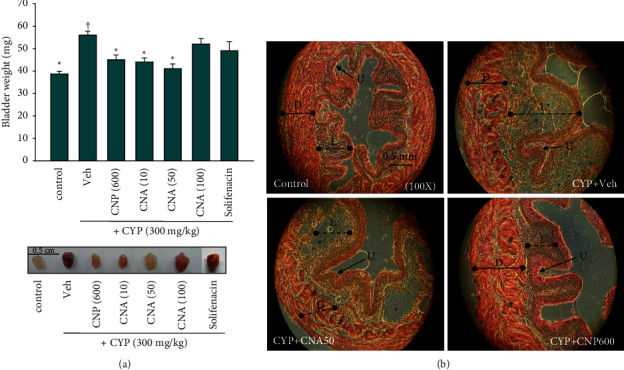
Edema in the bladder and hemorrhagic cystitis of cyclophosphamide-induced OAB in mice. Male *ICR mice* were subjected to cyclophosphamide- (CYP-, 300 mg/kg) induced OAB in the presence or absence of CNP and CNA. (a) Upper panel, edema in the bladder scored as weight (mg) changes was calculated and analyzed; lower panel, the degree of hemorrhagic cystitis was analyzed and scored in Table 1; (b) typical histological images (H&E stain) under microscope (100X); bladder was significantly swollen in the CYP + Veh group as compared with control; detrusor (D), lamina propria (L), and urothelium (U) were indicated. Tissues disordered with fragmentation (as hashtags indicated) and swollen with large empty spaces (as stars indicated) in the lamina propria and detrusor were clearly observed in the CYP + Veh group and scored in Table 1. Animal groups: (1) sham control group (control, *n* = 5) without CYP induction (control); (2) vehicle (normal saline with 0.1% of dimethyl sulfoxide (DMSO)) group with CYP (CYP + Veh, *n* = 5); (3) one CNP (at 600 mg/kg, 30 min after CYP induction, p.o.) treatment group with CYP (CYP + CNP600, *n* = 5); and (4) one CNA (at 50 mg/kg, 30 min after CYP induction, p.o.) treatment group with CYP (CYP + CNA5, *n* = 5).

**Figure 4:**
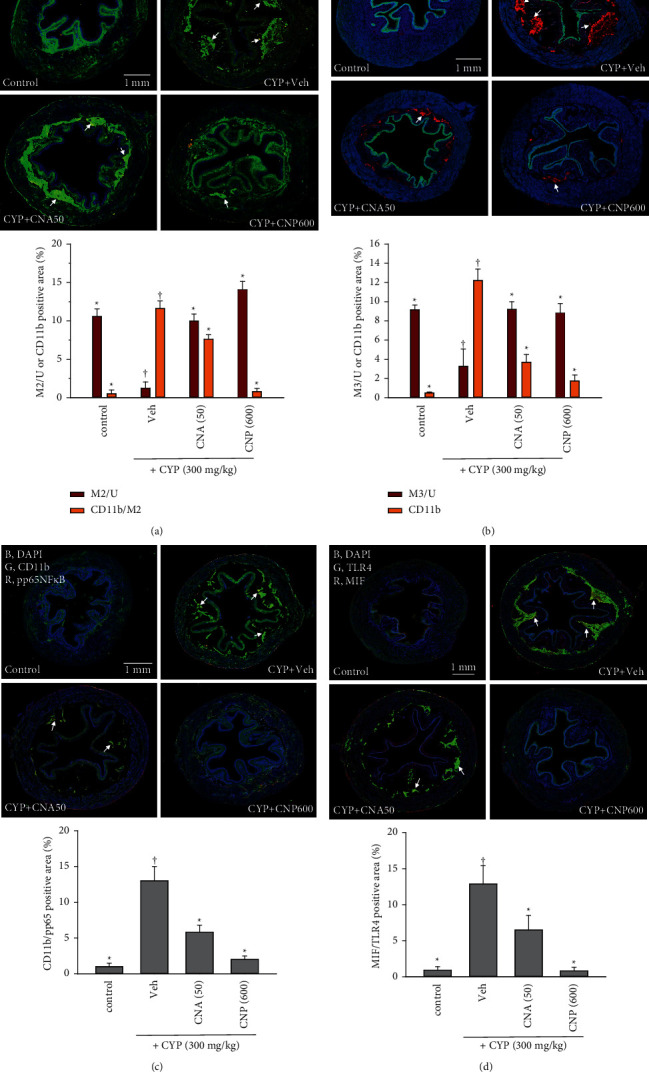
Effects of CNP and CNA on changes in markers of muscarinic receptors (M2 and M3), inflammation (CD11b), and inflammatory signaling proteins (pp65 NF*κ*B, TLR4, and MIF) in cyclophosphamide- (CYP-, 300 mg/kg) induced OAB mice 24 hours after CYP induction. Typical confocal images from bladder slices of animal groups described in Figure 3 show changes in (a) M2 (green, G), (b) M3 (green, G), and CD11b (red, R), and DAPI (blue, B) (a marker for nuclei), (c) colocalization of CD11b (green, G) with pp65NF*κ*B (red, R), and (d) colocalization of TLR4 (green, G) with MIF (red, R). Arrows indicate leukocyte (CD11b) colocalization with M2 (A), CD11b infiltration within lamina propria around M3 (B), and CD11b colocalized with pp65NF*κ*B (C), or colocalization of TLR4 with MIF (D). Statistical results of positive stain area (%) of whole image, such as M2 or M3 in urothelium (M2/U or M3/U) or colocalization (%) of CD11b/pp65NF*κ*B and MIF/TLR4 of whole images. Data are presented as mean ± SEM (*n* = 5). ^†^^*∗*^*p* < 0.05, compared with control or CYP + Veh group, respectively, by one-way ANOVA followed by S-N-K post hoc *t*-test for multiple comparisons.

To examine whether administration of CNP and CNA could improve CYP-induced tissue damage, histopathological examinations of bladder tissue after acute CYP-induced OAB in mice were performed. Inspection of the bladder showed dramatic tissue edema (weight increase, Figure 3(a) upper, *p* < 0.05) and hemorrhagic cystitis (Figure 3(a) lower and Table 1, *p* < 0.05) in the CYP + Veh group. These changes were significantly improved in the CYP + CNA50 and CYP + CNP600 groups, but not in the CYP + solifenacin group (Figure 3(a) and Table 1, *p* < 0.05). H&E staining of bladder sections from the control group revealed typical histologic architecture (Figure 3(b)). It was composed of three main layers, including an inner urothelial lining (urothelium, U) with normal corrugated folds and a collagen-rich vascularized lamina propria (L) middle layer surrounded by a highly compliant detrusor (D) smooth muscle interlaced outer layer (Figure 3(b), control). However, the corrugated folds and thickness of urothelium were significantly decreased in the CYP + Veh group (Figures 3(b) and 4(a), reduced staining of DAPI) but largely preserved in the CYP + CNA50 and CYP + CNP600 groups (Figures 3(b) and 4(a)). The lamina propria (L) displayed severe edema (swollen with large empty spaces) in the CYP + Veh group but moderate edema in the CYP + CNA50 group (Figure 3(b), enlarged interval of (L), around 1.10-fold (moderate) to 2.25-fold (severe) of control); in contrast, the CYP + CNP600 group presented slight edema in the lamina propria with an interval of (L) comparable to that of control group (Figure 3(b)). In the CYP + Veh group, the detrusor muscle layers formed many empty spaces between the fibers and were arranged in disorder with fragmentation. This could have been due to edema-induced tissue damage. However, these histopathological changes were significantly less extensive in the CYP + CNA50 and CYP + CNP600 groups (Figure 3(b) and Table 1, *p* < 0.05), but not in the solifenacin treatment group (Table 1, *p* > 0.05).

### 3.3. Effects of CNP and CNA on Changes in the Expression of Muscarinic Receptors (M2 and M3) and CD11b/pp65 NF*κ*B-Mediated Inflammatory Responses

The muscarinic receptors, especially M2 and M3, mediate the control of micturition function in the bladder [25, 26]. To examine whether muscarinic receptors could be damaged due to inflammatory responses in CYP-induced OAB, immunohistochemical staining was used. Results showed that severe and diffused inflammation characterized by leukocyte infiltration (CD11b) in the lamina propria and accompanied by reduced expression levels of muscarinic M2 and M3 in the urothelium was noticed in the CYP + Veh group (Figures 4(a) and 4(b), *p* < 0.05). Furthermore, leukocyte infiltration (CD11b) was colocalized with the expression of an active-form, inflammatory transcription factor pp65 NF*κ*B (Figure 4(c), *p* < 0.05). These changes were significantly suppressed in mice administered with CNP and CNA (Figures 4(a)–4(c), *p* < 0.05).

### 3.4. Effects of CNP and CNA on Changes in MIF/TLR4-Mediated Inflammatory Signaling Pathway

MIF and TLR4 signaling mediates cyclophosphamide induced bladder injury [27, 28]. Here, CYP-induced severe damage to the bladder was accompanied by overexpression of MIF and its downstream signal TLR4 (Figure 4(d), *p* < 0.05), which was closely associated with inflammation injury (CD11b and pp65 NF*κ*B) (Figures 4(a)–4(c)). However, oral treatment with CNP (600 mg/kg) and CNA (50 mg/kg) 30 min after CYP induction both significantly inhibited overexpression of these signals (MIF and TLR4) (Figure 4(d), *p* < 0.05).

### 3.5. Effects of CNP and CNA on Changes in SCF/c-Kit-Mediated Fibrotic Pathway

**Figure 5:**
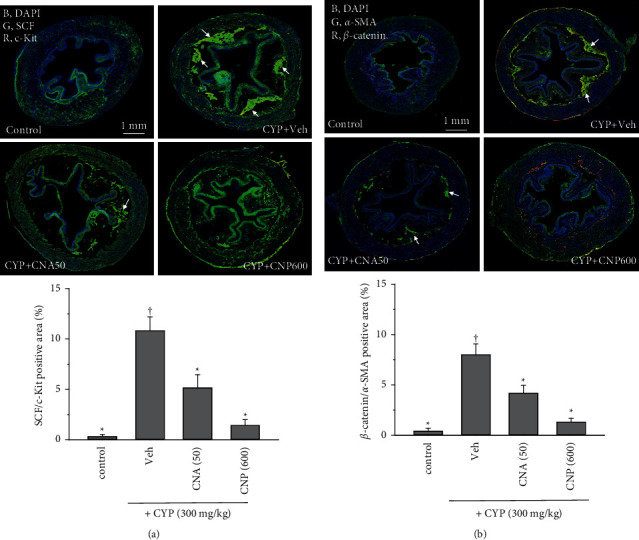
Effects of CNP and CNA on changes in the signaling of tissue fibrosis including SCF/c-Kit and *β*-catenin/*α*-SMA in cyclophosphamide- (CYP-, 300 mg/kg) induced OAB mice 24 hours after CYP induction. Typical confocal images from bladder slices of animal groups described in Figure 3 show changes in colocalization of (a) SCF (green, G) with *c*-Kit (red, R), (b) *β*-catenin (red, R) with *α*-SMA (green, G) and DAPI (blue, B) (a marker for nuclei). Arrows indicate colocalization of SCF/*c*-Kit and *β*-catenin/*α*-SMA. Statistical results of colocalization stain positive area (%) of the whole image. Data are presented as mean ± SEM (*n* = 5). ^†^^*∗*^*p* < 0.05, compared with control or CYP + Veh group, respectively, by one-way ANOVA followed by S-N-K post hoc *t*-test for multiple comparisons.

He and his colleagues reported that the SCF/c-Kit signaling pathway mediated bladder injury in a diphtheria-pertussis-tetanus vaccine tissue fibrosis model [29]. Here, CYP-induced damage to the bladder muscarinic receptors (M2, M3) and inflammation (CD11b, pp65NF*κ*B) were accompanied by overexpression of SCF/c-Kit (Figure 5(a), *p* < 0.05) as well as its downstream signals *β*-catenin and *α*-SMA (Figure 5(b), *p* < 0.05). Oral treatment with CNP (600 mg/kg) and CNA (50 mg/kg) 30 min after CYP induction significantly inhibited overexpression of these fibrotic signals (SCF/c-Kit and *β*-catenin/*α*-SMA) (Figure 5, *p* < 0.05).

### 3.6. Effects of CNP and CNA on Changes in Protein Expression Levels of Some Selected Markers

**Figure 6:**
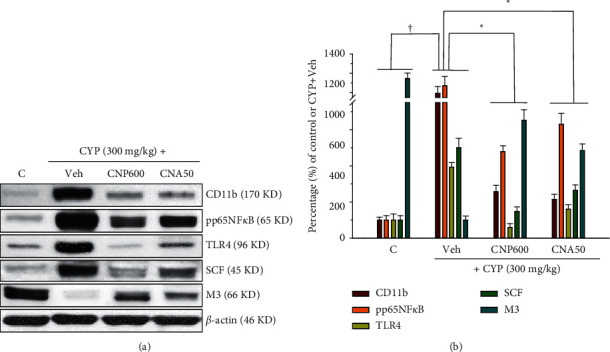
Effects of CNP and CNA on changes in selected protein expression levels of cyclophosphamide- (CYP-, 300 mg/kg) induced OAB mice 24 hours after CYP induction. (a) Upper, typical Western blotting analysis using whole bladder tissue from different groups was conducted to show changes in CD11b, pp65NF*κ*B, TLR4, SCF, and M3; *β*-actin was included for normalization. (b) Statistical results from the densitometry measurements after normalization to *β*-actin. Animal groups are as described in Figure 3. Data are presented as mean ± SEM (*n* = 5 for each group). ^†^^*∗*^*p* < 0.05, compared with the corresponding vehicle- (Veh-) treated OAB group (CYP + Veh) or sham control group (C), respectively, by one-way ANOVA followed by S-N-K *t*-test.

Furthermore, the protein expression levels of selected markers, namely, CD11b, pp65 NF*κ*B, TLR4, SCF, and M3, were examined by Western blotting for further comparison with IHC staining. Our data showed that inflammation-associated signals (CD11b, pp65 NF*κ*B, and TLR4) and fibrosis-associated signal (SCF) were dramatically increased, while the protein-mediated micturition function (M3) was significantly reduced in the CYP + Veh group (Figure 6, *p* < 0.05). However, oral treatment with CNP (600 mg/kg) and CNA (50 mg/kg) 30 min after CYP induction significantly inhibited inflammation- and fibrosis-associated signals and enhanced micturition function-related protein M3 (Figure 6, *p* < 0.05). These results were comparable to those observed in IHC staining (Figures 4 and 5).

## 4. Discussion

CYP-induced detrusor overactivity has been reported to cause decreased bladder capacity and increased micturition frequency [30], and it is a popular animal model used for OAB studies [21, 31, 32]. We found that CYP caused significant hemorrhagic cystitis, bladder inflammation with edema, and changes in micturition volume and frequency through inflammatory- and fibrotic-mediated signaling pathways, as revealed in previous studies [21, 29, 33, 34]. In addition, CNA has been reported to be a TRPA1 (transient receptor potential channel for ankyrin 1) agonist [17, 35]. TRPA1 is a cation channel receptor expressed in sensory nerves that innervate the rodent bladder and in the epithelium of both rat and human bladders [36]. It has been reported that treatment with a TRPA1 antagonist (HC-030031) can decrease the number and the amplitude of nonvoiding contractions (NVCs), an important parameter associated with the OAB etiology, induced by spinal cord injury (SCI) [37]; it was also found that SCI-caused bladder tissue damage, inflammation, and increases in the number of NVCs were associated with upregulation of TRPA1 protein and mRNA levels [37]. Furthermore, in another study, treatment with HC-030031 either before (100 mg/kg, p.o.) or after (30 mg/kg, i.v.) CYP (200 mg/kg, i.p.) inhibited the NVCs but failed to counteract the loss in voiding efficiency, leaving the TRPA1 expression level undetermined [38].

However, our supplementary data showed that administration of HC-030031 (100 mg/kg, 30 min after CYP once daily, p.o.) did not cause significant improvement in CYP-induced abnormal micturition patterns (*p* > 0.05, Figures S1–S3). Additionally, using immunohistochemical staining, we found that CYP-induced OAB was accompanied by a significant decrease of TRPA1 expression in the urothelium (Figure S4) as well as in the total protein expression level (Figure S5). In contrast, TRPA1 expression level was preserved by CNP and CNA (Figures S4 and S5), but administration of HC-030031 did not significantly reverse the protective effects of CNP and CNA (data not shown). Based on these observations, we propose that the preservation of TRPA1 expression was a beneficial effect achieved by CNP and CNA treatment, and TRPA1 activation was not directly involved in the OAB ameliorative effect of CNP and CNA in this model.

Furthermore, the bladder toxicity induced by CYP has been attributed to its metabolite acrolein, which, if not scavenged, damages the bladder, leading to hemorrhagic cystitis and TRPA1-dependent visceral pain [39]. However, it has been suggested that the main mechanism by which acrolein causes bladder damage is through the production of reactive oxygen species and nitric oxide, resulting in lipid peroxidation, protein oxidation and DNA damage, and depletion of nicotinamide adenine dinucleotide and ATP, ultimately causing necrotic cell death [40]. Therefore, it is reasonable to note that in this study, pretreatment with a TRPA1 antagonist (HC-030031) did not significantly reverse the CYP-mediated tissue damages.

In a double-blind, randomized, and placebo-controlled trial, we recently found that treatment with CNP patches could successfully alleviate the symptoms of OAB in patients without significant side effects [19]. The present study showed the first evidence that oral administration of either CNP or CNA can ameliorate CYP-induced detrusor overactivity and related pathophysiological changes, particularly preservation of muscarinic M2 and M3 receptors, most possibly by inhibiting the inflammatory- (MIF/TLR4) and fibrotic- (SCF/c-Kit) mediated signaling pathways. Regarding the mechanism(s) of action of CNP used in this study, comprising approximately 0.5% CNA, CNP displayed a better OAB-ameliorating effect than that of CNA. We propose that this could be attributed to the multiple components and diverse pharmacological effects of CNP [15]. In addition to the above mechanisms, CNA has the potential to treat stress urinary incontinence (SUI). SUI is a common disease in middle-aged women and the elderly. CNA may regulate a variety of SUI-related proteins, including myosin, inducible nitric oxide synthase, survival motor neuron protein, and superoxide dismutase 3 [15].

In TCM, Ba-Wei-Die-Huang-Wan (BWDHW) is used to treat diabetes and urinary frequency [41]. BWDHW contains eight ingredients, including cinnamon (3.7%). Treatment with BWDHW can reduce CYP-induced persistent overactive bladder in rats and inhibit the overexpression of mucosal purinergic (P2X2, P2X3) and muscarinic (M2, M3) receptors, as well as overexpression of muscarinic (M2, M3) receptor in detrusor [41]. However, our data show that CYP dramatically reduced muscarinic (M2, M3) receptor protein expression, typically within the urothelium, due to damage to the normal corrugated folds of the urothelium by CYP. This result was consistent with the strong staining of these receptors (M2, M3) colocalized with inflammatory markers (CD11b and pp65 NF*κ*B) in the damaged area (especially within the lamina propria) by CYP. Administration with CNP and CNA could effectively preserve these receptors (M2 and M3) in our model. This result could be further explained by the observation that administration with solifenacin, a M3 muscarinic receptor antagonist, did not significantly improve the bladder capacity or micturition frequency, nor did it reduce tissue edema or cystitis in this model. Since most of the total M2 and M3 receptors were diminished by CYP-induced inflammation and fibrosis in this model, solifenacin was not effective in reversing CYP-induced OAB syndrome and inflammation/fibrosis of the bladder by blocking M3 receptors alone.

Furthermore, the discrepancy between Lee et al.'s results [41] and ours can be attributed to the different animal species (rats vs. mice), CYP dosage (100 mg/kg vs. 300 mg/kg), drug treatment (eight herbs at 90 mg/kg/day for 5 days vs. single herb (CNP) at 600 mg/kg/day for 1 day), day(s) of analysis after CYP challenge (on day 7 vs. day 1), and analysis method (Western blotting after microdissection of the different tissue vs. confocal IHC imaging plus Western blotting analysis using whole bladder tissue slices). The regular dosage of CNP used in adult patients is between 1.5 g/day and 3.0 g/day. Here, we used the high dosage of CNP (3.0 g/day for human, i.e., around 600 mg/kg/day for mouse), which was sufficient to show the therapeutic effects. Further studies are needed to explore the significant implication of different herbal treatments (multiple vs. single ingredient) between these two models (subacute vs. acute).

In addition to the inhibition of inflammatory- and fibrotic-mediated signaling pathways, which could account for the protective effects of CNP and CNA, a drug with antioxidative potential, such as resveratrol, can improve urinary dysfunction in rats with chronic prostatitis and suppress the SCF/c-Kit signaling pathway activity [42]. Resveratrol also improves detrusor fibrosis induced by mast cells during the progression of chronic prostatitis in rats [29]. In this study, we found that neither CNP nor CNA displayed free radical (DPPH) scavenging activity, nor did they modulate the expression of nuclear factor erythroid 2-related factor 2 (*Nrf2*), a novel regulator of cellular resistance to oxidants (data not shown).

## 5. Conclusions

**Figure 7:**
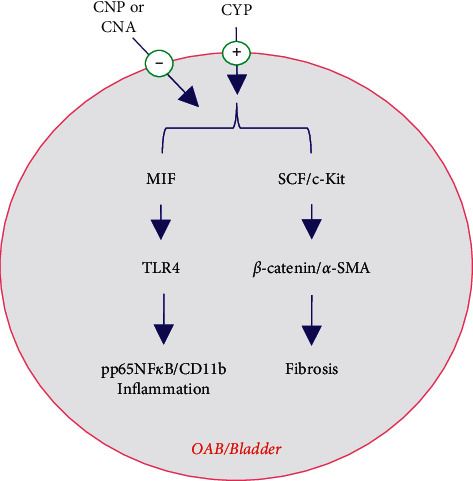
Graphical illustration showing the key pathways regulated by CNP and CNA. CNP and CNA have a profound effect on the inhibition of cyclophosphamide- (CYP-) induced OAB bladder injury in inflammation and fibrosis pathways *via* inhibiting MIF/TLR4- and SCF/c-Kit-mediated signal transduction, which in turn suppresses pp65NF*κ*B/CD11b and *β*-catenin/*α*-SMA upregulation, respectively.

In summary, our results showed that treatment with CNP and CNA could inhibit MIF/TLR4-associated inflammatory and SCF/c-Kit-related fibrotic pathways and thereby ameliorate CYP-induced OAB (Figure 7). Based on our recently published clinical trial and the findings of this study, we strongly recommend that CNP and CNA be considered as useful choices for OAB treatment in the clinic.

## Figures and Tables

**Table 1:** Summary of histological changes in bladder in the CYP-induced OAB experimental groups.

	Control^*∗*^	CYP + Veh^†^	CYP + CNP^*∗*^	CYP + CNA^*∗*^	CYP + Soli.
Hem. C.	0.0 ± 0.0	3.0 ± 0.0	0.8 ± 0.2	0.4 ± 0.2	2.5 ± 0.4
D. & F.	0.0 ± 0.0	3.0 ± 0.0	0.8 ± 0.2	1.6 ± 0.2	2.2 ± 0.2

Tissue structure of the bladder arranged in disorder with fragmentation and empty spaces (D. & F.) in damaged areas including detrusor (D), lamina propria (L), and urothelium (U), and hemorrhage cystitis (Hem. C.) (Figure 3) were scored as negative (0), slight (1), moderate (2), and severe (3) by three investigators who were blind to animal groups. Animal groups: (1) a sham control group without cyclophosphamide (CYP) induction (control); (2) cinnamon powder (CNP) (at 600 mg/kg, 30 min after CYP induction, p.o.) treatment group with CYP (CYP + CNP); (3) cinnamaldehyde (CNA) (at 50 mg/kg, 30 min after CYP induction, p.o.) treatment group with CYP (CYP + CNA); (4) solifenacin (at 50 mg/kg, 30 min after CYP, p.o.) treatment group with CYP (CYP + Soli.); and (5) vehicle (normal saline with 0.1% of dimethyl sulfoxide (DMSO)) group with CYP (CYP + Veh). Data are presented as mean ± SEM (*n* = 5). ^†^, ^*∗*^*p* < 0.05, compared with control or CYP + Veh, respectively, by one-way ANOVA followed by S-N-K post hoc *t*-test for multiple comparisons.

## Data Availability

The data used and analyzed in this study are available from the corresponding author on reasonable request.

## Abbreviations

*α*-SMA: *α*-Smooth muscle actin
*c*-Kit: An SCF receptor
CNA: Cinnamaldehyde
CNP: Cinnamon powder
CYP: Cyclophosphamide
MIF: Macrophage migration inhibitory factor
OAB: Overactive bladder
SCF: Stem cell factor
TLR4: Toll-like receptor 4.

## Supplementary Materials

Supplementary Materials Materials (HC-030031 and TRPA1 (antibody)) used in the supplementary data (Figures S1–S5) are included in the Materials and Methods section.
