# Supplementary figures and images for: Suppression of Inflammatory and Fibrotic Signals by Cinnamon (Cinnamomum cassia) and Cinnamaldehyde in Cyclophosphamide-Induced Overactive Bladder in Mice
Source: Evid Based Complement Alternat Med. 2021 Dec 22;2021:5205759. doi: 10.1155/2021/5205759 (PMC8716214; doi:10.1155/2021/5205759)

## Supplementary Data

Fig. S1

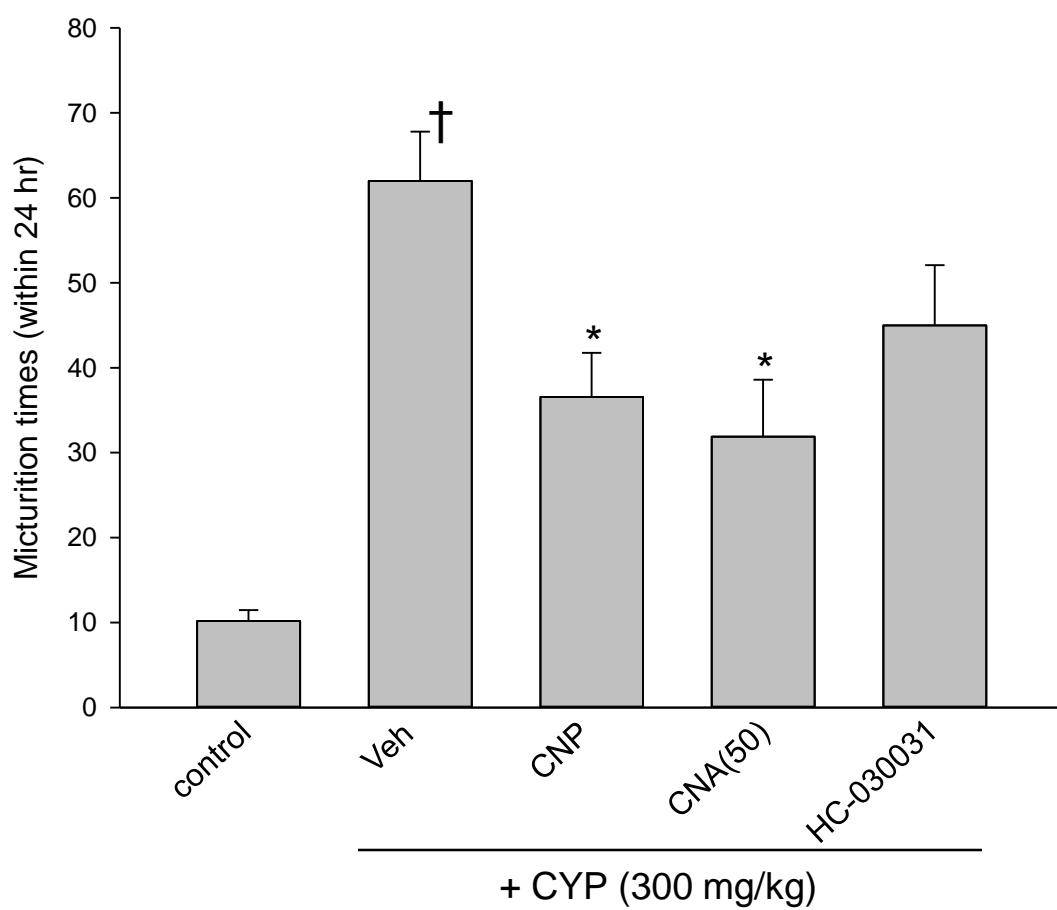

Fig. S2

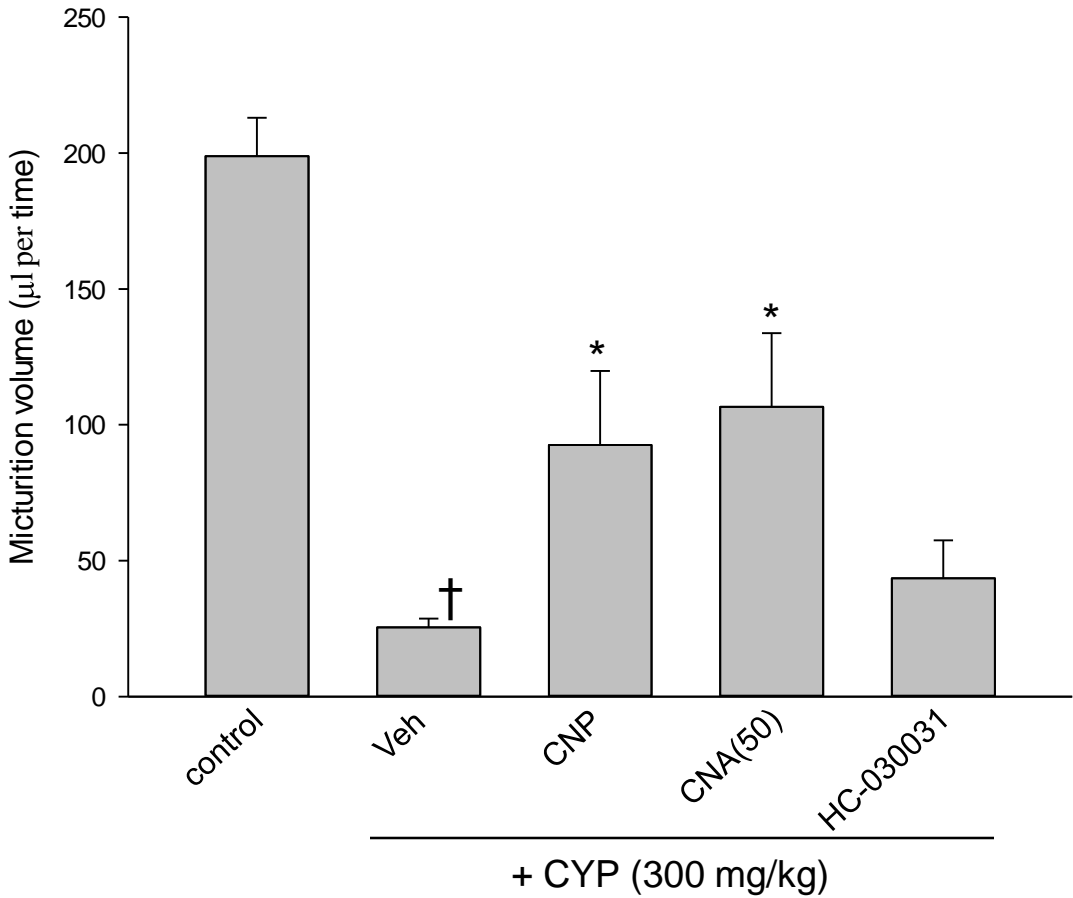

Fig. S3

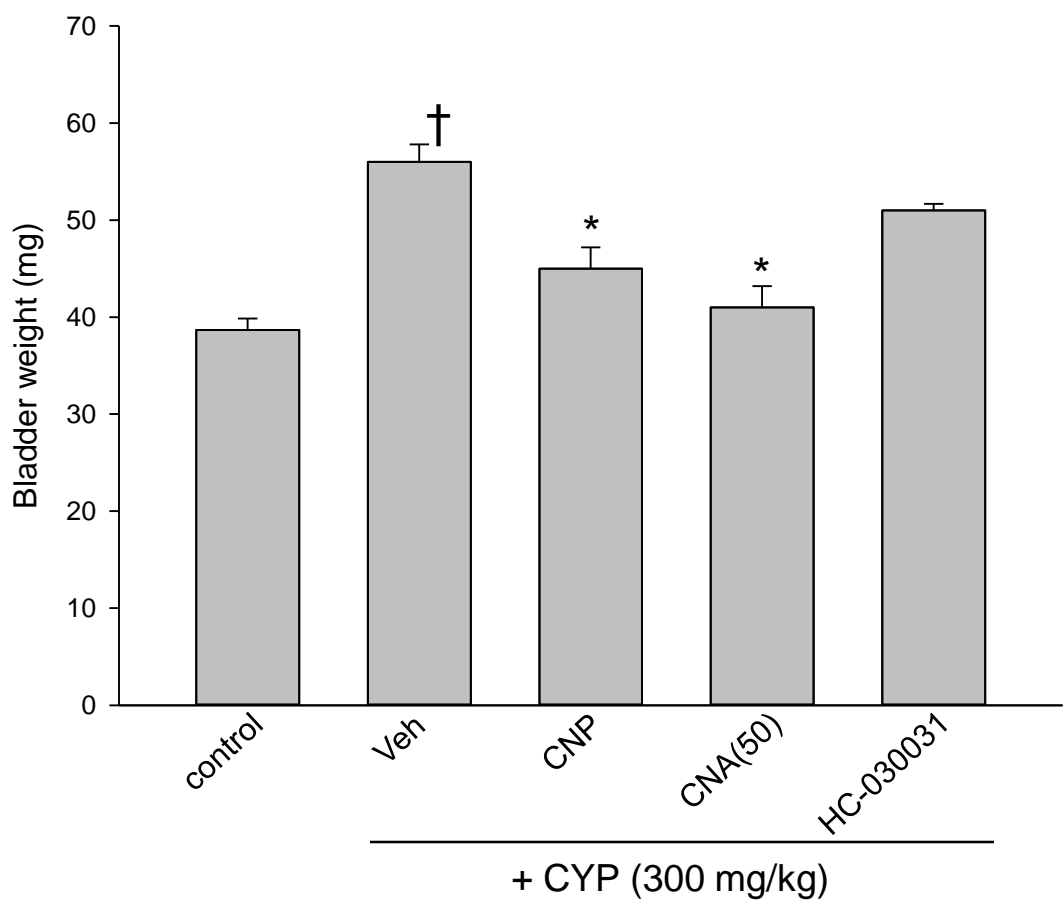

Fig. S4

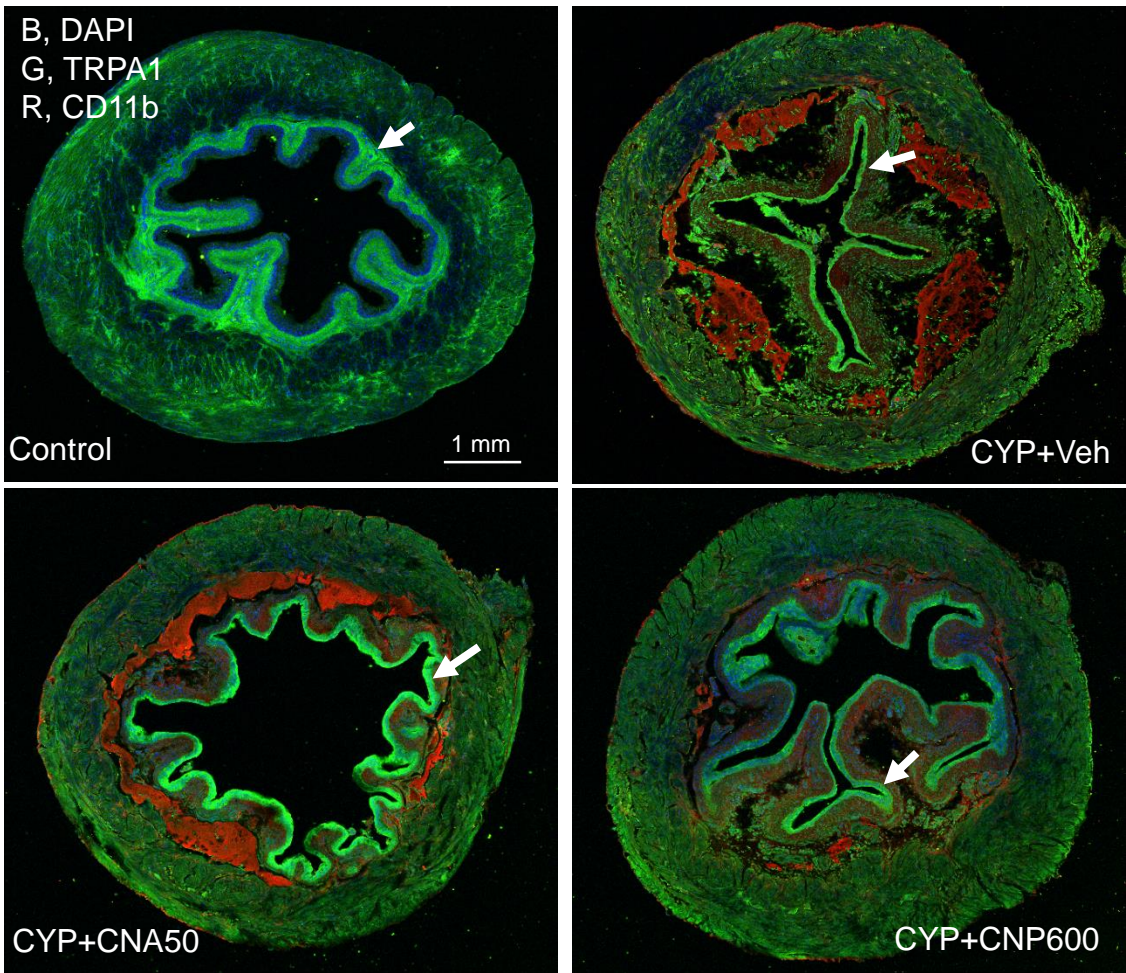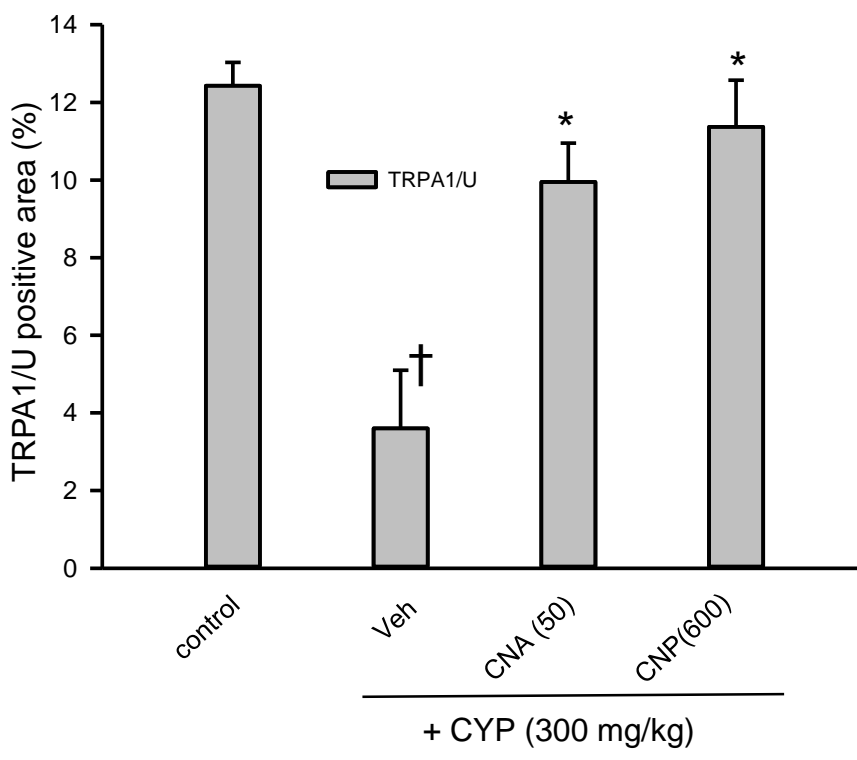

Fig. S5

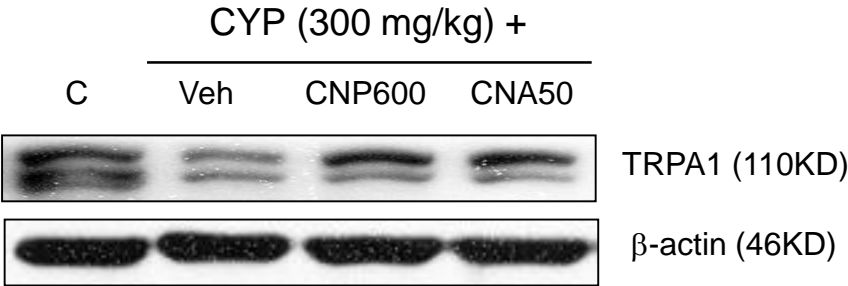

Supplement: Supplementary Materials — Materials (HC-030031 and TRPA1 (antibody)) used in the supplementary data (Figures S1–S5) are included in the Materials and Methods section. [file 5205759.f1.pdf]
